# Computational Design of New Peptide Inhibitors for Amyloid Beta (Aβ) Aggregation in Alzheimer's Disease: Application of a Novel Methodology

**DOI:** 10.1371/journal.pone.0066178

**Published:** 2013-06-06

**Authors:** Gözde Eskici, Mert Gur

**Affiliations:** 1 Center for Computational Biology and Bioinformatics, Koc University, Istanbul, Turkey; University of Akron, United States of America

## Abstract

Alzheimer's disease is the most common form of dementia. It is a neurodegenerative and incurable disease that is associated with the tight packing of amyloid fibrils. This packing is facilitated by the compatibility of the ridges and grooves on the amyloid surface. The GxMxG motif is the major factor creating the compatibility between two amyloid surfaces, making it an important target for the design of amyloid aggregation inhibitors. In this study, a peptide, experimentally proven to bind Aβ40 fibrils at the GxMxG motif, was mutated by a novel methodology that systematically replaces amino acids with residues that share similar chemical characteristics and subsequently assesses the energetic favorability of these mutations by docking. Successive mutations are combined and reassessed via docking to a desired level of refinement. This methodology is both fast and efficient in providing potential inhibitors. Its efficiency lies in the fact that it does not perform all possible combinations of mutations, therefore decreasing the computational time drastically. The binding free energies of the experimentally studied reference peptide and its three top scoring derivatives were evaluated as a final assessment/valuation. The potential of mean forces (PMFs) were calculated by applying the Jarzynski‚s equality to results of steered molecular dynamics simulations. For all of the top scoring derivatives, the PMFs showed higher binding free energies than the reference peptide substantiating the usage of the introduced methodology to drug design.

## Introduction

Amyloidosis is an extracellular accumulation of insoluble protein fibrils in an abnormal form.[1] Amyloids, which are the aggregates formed by the self-association of such insoluble protein fibrils, are associated with serious neurodegenerative and prion diseases including Alzheimer's disease, type 2 diabetes, Parkinson's disease, and Huntington's disease.[2] Knowing how these amyloids form stable structures is essential for the design of effective therapeutic molecules.

**Figure 1:**
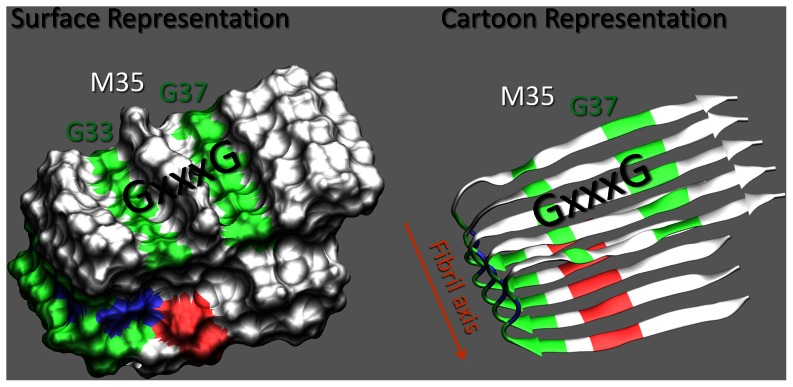
Structure of Protofilament Subunit of Aβ42. The Amyloid fibril (PDB ID: 2BEG) is shown in two separate representations; (i) Molecules are drawn as surfaces and (ii) molecules are drawn as secondary structure cartoons. Coloring is performed according to the residue type (non-polar residues (white), basic residues (blue), acidic residues (red) and polar residues (green)). Images were rendered using VMD.[38]

Amyloid fibrils have characteristic spatial organizations (shown in Fig.1), forming cross β-sheet structures by the association of β-strands.[3] The term cross-β fibril refers to the overall structure where individual β strands are arranged in a parallel, in-register form.[4] Physical, biomolecule based, and chemical strategies have been developed to intervene and inhibit the formation of amyloidosis (Recently reviewed by Liu et al.[5] and Hard et al. [6] ). Biomolecule based and chemical strategies can be categorized according to how they intervene/inhibit amyloid formation, such as (i) proteins or small molecules that bind and stabilize a native folded state of a protein, (ii) proteins that bind to aggregation-prone regions of amyloidogenic peptides and prohibit self assembly (sequester monomers from aggregation), (iii) small-molecules that target the misfolding and aggregation of proteins (e.g. counteract self assembly of amyloidogenic proteins ), (iv) peptide-based inhibitors of amyloid growth and/or (v) antibody-mediated inhibition and immunotherapy.[6]

The *peptide-based inhibitors of amyloid growth* strategy (reviewed by Sciaretta et al.[7]) has drawn much attention in the last two decades. Numerous peptide fragments were designed to bind critical regions for aggregation on the beta-amyloid proteins and, by doing so, inhibit amyloid aggregation [5], [8], [9], [10], [11]. These peptides either bind to the Aβ surface and prevent fibrillization, or interfere with elongation in the fibril axis (Fig.1) direction by binding to monomers or to oligomers. Three consecutive repeats of the GxxxG motif encompassing Aβ residues Gly33 to Gly37 form molecular ridges and grooves on the amyloid surface.[12] These ridges and grooves were proposed [12] to facilitate amyloid fibril aggregation and be critical for the rational design of inhibitors to prevent fibril aggregation. A model peptide (GpA70–86) composed of spanning residues of the transmembrane helix of glycophorin A was studied experimentally by Liu et al.[13] to reveal the role of glycine and the importance of the GxxxG motif. Their study showed that the amino acids with large side chains form molecular ridges which can fit into the glycine grooves, GxxxG, and such compatibility between both surfaces stabilizes amyloid fibril formation.

Liu et al. [13] have successfully designed an 8-residue peptide, RGTFEGKF-NH_2_, that breaks the compatibility between two amyloid fibril surfaces by targeting their glycine grooves. The inhibitor (RGTFEGKF-NH_2_) was designed so that the small residue glycine alternates with the bulky residue phenylalanine on one face of the peptide, xGxFxGxF, whereas the polar and charged residues were placed on the opposite face of the peptide as RxTxExKx. The xGxFxGxF sequence was selected to be complementary to the GxMxG sequence in the C-terminus of Aβ42 and RxTxExKx gave the peptide its solubility. Experiments demonstrated that RGTFEGKF derivatives were also effective in the inhibition of Aβ fibrilogenesis.[12] In addition, different peptides that varies 9 to 12 residue in length have been shown to be effective in binding critical regions and preventing oligomerization of or fibrilization of Aβ protein.[14], [15], [16], [17]

In this study we generate a small library of peptide inhibitor candidates by systematically mutating the residues of RGTFEGKF. Residues are first replaced one at a time with ones that share similar chemical characteristics and mutations are assessed via docking whether they increase the binding affinity of the peptide to Aβ42 relative to the original peptide. Mutations that increase the docking score are combined and combinations are reevaluated using docking until the peptide library attains a desired number of inhibitors. The final peptide library contained 300 peptides with up to 4 mutations per peptide. Rescoring with a different scoring function decreased the size of the peptide library to 11 peptides. The three top scoring candidate inhibitors were further assessed by performing a total of 600 ns of unbinding simulations from the amyloid protofilament subunit in explicit solvent via steered molecular dynamics (SMD) and subsequently generating their potential of mean forces (PMFs) using Jarzynski's equality. All of these 3 mutated peptides showed higher binding free energies than the original peptide, validating the usage of our methodology to generate new inhibitor candidates.

## Materials and Methods

### Restricting Mutations

If every possible derivative of the reference peptide is to be generated, each amino acid will have to be exchanged with all the other 19 amino acids, ending up with 20^8^ different sequences. Since handling such a large number of sequences is burdensome, this number was reduced by restricting the allowed modifications. The sequence of the reference peptide (inh) was mutated by replacing its amino acids only with ones belonging to the same amino acid group. This grouping is based on the general chemical properties of their side chains as shown in Table 1. In addition, glycine was not mutated into methionine because the methionine derivative of the reference peptide was observed not to be effective.[12] For example, Arg1 was mutated into His and Lys, which are positively charged at or around physiological pH, resulting in the sequences HGTFEGKF and KGTFEGKF. Similarly, Lys at the seventh position was replaced with Arg and His, resulting in the sequences RGTFEGRF and RGTFEGHF. Peptides that differ from the reference peptide by only one residue in sequence will be referred to as one-point mutated peptides. Similarly, the ones that differ by two, three and four residues will be referred to as two-, three- and four-point mutated peptides.

**Table 1:** Grouping System and Hydrophobicity Index[39] Used in This Study.

**Nonpolar, Aliphatic**			**Polar, uncharged**		
Glycine	Gly G	−0.4	Serine	Ser S	−0.8
Alanine	Ala A	1.8	Threonine	Thr T	−0.7
Valine	Val V	4.2	Cysteine	Cys C	2.5
Proline	Pro P	1.6	Asparagine	Asn N	−3.5
Leucine	Leu L	3.8	Glutamine	Gln Q	−3.5
Isoleucine	Ile I	4.5	**Negatively charged**		
Methionine	Met M	1.9	Aspartate	Asp D	−3.5
			Glutamate	Glu E	−3.5
**Aromatic**			**Positively charged**		
Phenylalanine	Phe F	2.8	Lysine	Lys K	−3.9
Tyrosine	Tyr Y	−1.3	Histidine	His H	−3.2
Tryptophan	Trp W	−0.9	Arginine	Arg R	−4.5

### Combine, Mutate and Dock; Generating Potential Inhibitor Candidates

**Figure 2:**
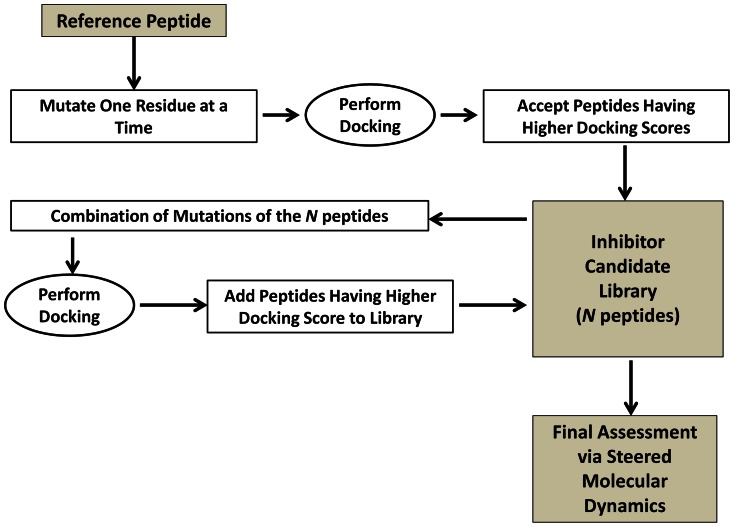
Flowchart of the Methodology.

The methodology, schematically shown in Fig.2, is constructed of two main steps and a subsequent final assessment step;

#### (i) The Initiation of the Inhibitor Candidate Library

To initiate the methodology one residue at a time is mutated, resulting in 23 single-residue mutated peptides. The docking scores of these 23 one-point mutated peptides are calculated. Mutations that result in a higher binding score in comparison to the reference peptide are accepted. These *n*_1_ peptides are used to the initiate the inhibitor candidate library (ICL).

#### (ii) Recursive Combination of Mutations and Docking

The 2-combinations of all mutations in the ICL are performed and then assessed via docking. If combined new mutations result in a higher docking score than the reference peptide they are accepted and added to the ICL. If not they are simply rejected. This recursive cycle is repeated until a desired number inhibitor candidates or level of mutation is obtained.

For example, in the first cycle all single residue mutations of the *n*_1_ peptides in the ICL are combined. For these two-point mutated peptides, docking is performed and peptides resulting in higher binding scores than the reference peptide are accepted. These *n*_2_ number of two-point mutated peptides are added to the ICL, resulting in a library size of *N* = *n*_1_+*n*_2_ peptides. In the second cycle, the mutations of the *n*_1_ one-point mutated peptides and the *n*_2_ two-point mutated are combined. Docking is performed and peptides are accepted if docking score is higher than the reference peptide. The accepted *n*_3_ number of three-point mutated peptides and *n*_4_ number of four-point mutated peptides are appended to the ICL making the total library size *N* = *n*_1_+*n*_2_+*n*_3_+*n*_4_.

#### (iii) Final Assessment

Starting from their docked structures conventional molecular dynamics (CMD) simulations are performed for the top scoring inhibitor candidates. *In silico* unbinding experiments are performed on these ensembles (see the *Constructing the Potential of Mean Force* and *Molecular Dynamics simulation* sections for details) and binding free energies are calculated.

### Docking

Docking studies of the designed peptides is carried out using GOLD Genetic Optimization for Ligand Docking) 4.1 program from the Cambridge Crystallographic Data Center, UK. [18] GOLD uses a genetic algorithm for docking flexible ligands into a protein binding site to explore the full range of ligand conformational flexibility with the partial flexibility of the protein. Bound conformations are predicted and assessed using Goldscore. In their study Verdonk et al. [19] have shown that using a second scoring function to rescore dockings can give significant improvements in success rates compared to straightforward docking using just a single scoring function. Hence, dockings are rescored with Chemscore. Goldscore takes four factors into account; (i) The protein-ligand hydrogen bonding (ii) The protein-ligand van der Waals interactions (iii)The internal van der Waals energy of the ligand (This is switched off by Gold default) and (iv) The torsional strain energy of the ligand. Chemscore on the other hand estimates the total free energy upon ligand binding and was parameterized against the experimental binding affinities for a test set of 82 protein-ligand complexes.[19]

Aβ42 protofilament subunit (PDB ID: 2BEG) is used as the receptor for docking. This solution NMR structure comprises 5 Aβ strands each strand containing 26 residues. The ligand binding site in docking is defined as a collection of residues placed within a sphere of 20 Å diameters around the coordinates of M35 in the GxMxG motif (GLMVG) of the third Aβ-strand (Aβs3). In order to obtain a diverse set of high scoring docking conformations 100 different bound conformations are allowed to be tested and the early termination option is not used. All other parameters were kept at their default values. After visual inspection docked Protein-Peptide conformers where the Peptides did not fit into the glycine grooves were discarded.

### Constructing the Potential of Mean Force

Constant velocity SMD simulations [20], [21]_ENREF_13 were performed in which the center of mass of the backbone atoms of residues 4–5 of each peptide are attached to a dummy atom via a virtual spring with a spring constant of *k* = 5 kcal/(mol •Å). The backbone atoms of M35 (methionine in the GxMxG motif) in Aβs3 of the protein are fixed. The dummy atom is then pulled with a constant velocity along the reaction coordinate (RC), which is defined as the vector between the center of mass of the pulled peptide atoms and the fixed protein atoms. The RC, *ξ*(**r**), is a function of the 3N-dimensional position **r** of the system. The distance of the dummy atom along the RC, *λ*(*t*), changes with a constant velocity as 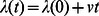, where 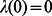. Hence, the external work is calculated as,[21]

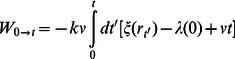
(1)


Jarzynski's equality is a relation between equilibrium free energy differences, Δ*A*, and work done through non-equilibrium processes, *W.* [21] Jarzynski's equality states that the following equality holds regardless of the speed of the process.[22], [23]

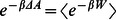
(2)


Where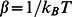, 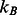
is the Boltzmann constant and *T* is the temperature. The average 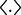
in Eq. 2 is taken over the ensemble of SMD trajectories whose starting structures are sampled from conventional MD simulation. The Jarzynski's equality is a methodology that evaluates the free energy differences between two points, which are defined by a parameterized quantity *λ,* via the work values along the paths that connect them. In this study λ is the dummy atom coordinate. To calculate the potential of mean force (PMF) of the original system, 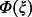, at a specified reaction coordinate, 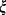, work values for different values of 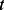 ( or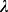 ) but being at the same 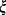 have to be combined. When the spring constant 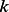 of the guiding potential is sufficiently large so that the reaction coordinate follows the constraint center 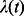 closely, the following stiff-spring approximation emerges[21]: 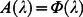
(3)


Hence, the PMF will be evaluated by the Jarzynski's equality using the work values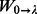. Due to the external potential applied to the SMD atoms the conformation of the peptides will be lightly biased and may not be in their equilibrium state. However, to relax these final states no external work is required and the final free energy differences are not affected.

### Molecular Dynamics simulations

Molecular dynamics (MD) simulations were performed in explicit solvent (water) using NAMD 2.8 [24] package with CHARMM27 [25] force field. Simulations were performed at 310 K temperature and 1 bar pressure. The RCs of the docked forms of the top scoring 3 peptides of the ICL and reference peptide were aligned with the positive x-axis. Each protein-peptide complex was then solvated in a water box with a 50 Å cushion in the positive x direction and 10 Å cushions in the other directions. Periodic boundary conditions were applied. Na^+^ and Cl^−^ ions were added into water to represent a more typical biological environment at 0.15M concentration and make the net charge of the system zero. All solvated and ionized systems had a total atom number slightly larger than 30,000 atoms. Langevin dynamics was used to control the temperature and Langevin piston Nose-Hoover method was used to control the pressure of the system. A time step of 1fs was used. Non-bonded and electrostatic forces were evaluated at each time step. Electrostatics are computed using particle mesh Ewald method. Van der Waals interactions are cut off beyond 12 Å and the switching function, which smoothly brings the forces and energies to 0 at the cutoff distance, started at 10Å. In order to keep all degrees of freedom, no rigid bonds were used. Two minimization-equilibration cycles were performed. The first cycle was performed under T, P, N conditions keeping the protein fixed in order to relax the water. The subsequent minimization to the first minimization-equilibration cycle was performed under T, V, N conditions with no constrains on the protein. After this final minimization, CMD simulations under T, V, N conditions were performed for 8 ns so that large ensembles of equilibrated conformations of the complexes were generated. Fourteen starting structures for the SMD simulations were sampled with 0.25ns intervals from the trajectory stretch 0.75–2.5ns of the CMD simulations. The subsequent parts of trajectories (2.5-8ns) were performed to show that the peptides stay bound during the time length of the SMD simulations.

Park et al. [21] have shown that for the unfolding of helical Deca-alanine in vacuum a pulling speed of resulted in a reversible process. In literature [26], [27], [28] a range of pulling velocities between and were successfully applied for the unbinding processes. In our work two different pulling velocities of and were applied. For each peptide four SMD simulations were performed with and ten SMD simulations were performed with . A total of 600 ns of SMD simulations were executed giving us an extensive data set.

## Results and Discussion

### Inhibitor Candidates

**Figure 3:**
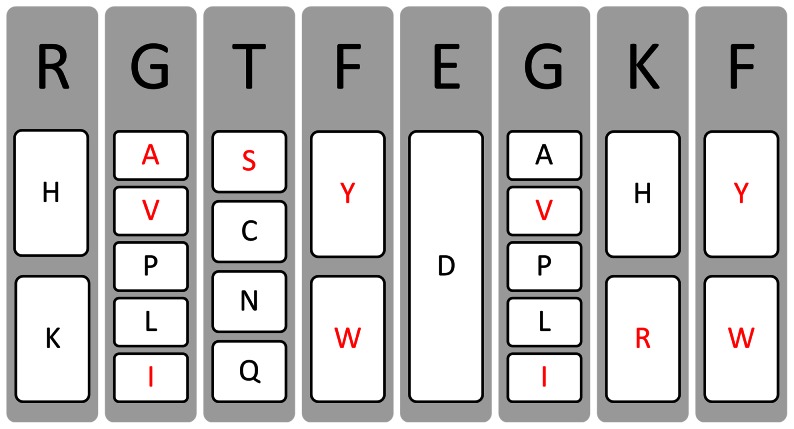
Single Residue Mutations. Tested single residue mutations based on grouping system. Residues shown in red indicate mutations that resulted in higher docking scores.

**Figure 4:**
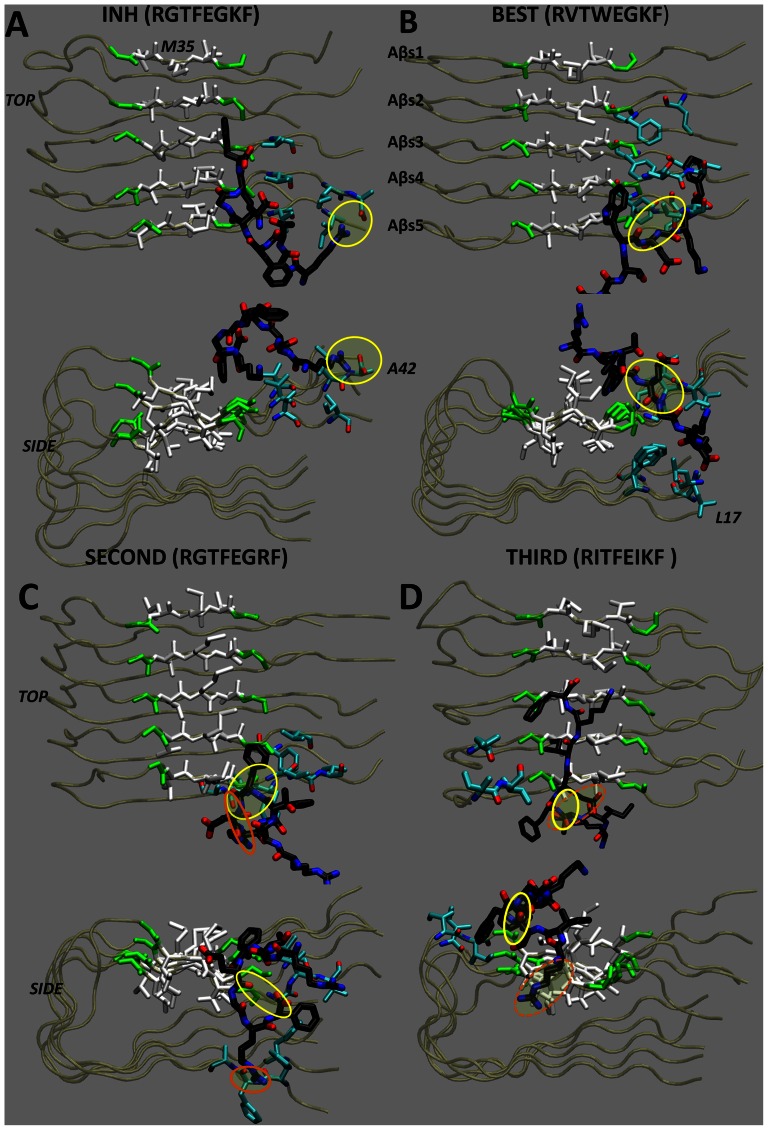
Bound Conformations of Inhibitor Candidates. Equilibrated bound conformers of the (a) reference peptide, (b) the top scoring peptide, RVTWEGKF, (c) the second highest scoring peptide, RGTFEGRF, and (d) the third best scoring peptide, RITFEIKF, to the Aβ42 fibril are shown. The conformations at time instant 2ns of the CMD are depicted. The protein is represented in transparent tube representation. The GxMxG motif is shown by its residue type. The peptide and all residues within 3.5Å of it are shown by the licorice drawing method. To distinguish the peptide and protein residues easily, peptide carbons were colored in black.

23 single-residue mutations were performed based on the grouping system provided in Table 1. Mutations are shown schematically in Fig. 3. 11 single residue mutations resulted in higher Goldscore docking scores than the reference peptide and are shown with red letters in the figure. The ICL was initialized with these 11 one-point mutated residues. The recursive combine-mutate-dock cycle was performed twice and mutations were accepted or rejected depending on their Goldscores. At the end of these two cycles the ICL contained *N* = 300 peptides. In order to further decrease this number, their Chemscore docking scores were evaluated. Among the 300 peptides, only 11 showed better Chemscores than the reference peptide. These11 peptides, which bind the amyloid surface with a better Chemscore and Goldscore values than the reference peptide, are listed in Table 2 together with these values. The binding conformations of the top scoring 3 peptides and the reference peptide are shown in their bound form in Fig. 4.

**Table 2:** Docking Scores of RGTFEGKF and its Derivatives.

No.	Sequence	Chemscore (kJ/mol)	Goldscore
(INH1) RGTFEGKF		−7.13	49.12
1.	RVTWEGKF	−15.01	67.56
2.	RGTFEGRF	−13.25	65.36
3.	RITFEIKF	−10.86	63.48
4.	RGTWEIKW	−10.39	52.02
5.	RGSWEGKF	−10.11	52.86
6.	RGSFEGKW	−10.04	52.45
7.	RGTWEVKF	−9.35	59.31
8.	RGSFEGKF	−8.95	53.07
9.	RVTWEVKF	−8.83	52.27
10.	RGTWEGKF	−8.83	49.87
11.	RGSWEGKW	−8.20	49.26

Mutated residues are underlined.

### Binding Characteristics of the Inhibitor Candidates

The reference peptide is anchored to the amyloid fibril surface by its arginine at the N-terminal and its phenylalanine at the C-terminal. The aromatic side chain of phenylalanine fits into the hydrophobic glycine groove where it has hydrophobic interactions mainly with M35s of Aβs2, Aβs3 and Aβs4. As shown in Fig.4 (a), the guanidinium group at the N-terminal (R1) of the peptide forms hydrogen bond with the backbone carboxyl group of the C-terminal alanine A42 of the fourth beta sheet. Electrostatic interactions between polar and charged residues, such as the side chain OH group of T3 and the side chain amine of K7, result in a packed hairpin-like conformation of the peptide, which limits interactions of residues 2-7 with the amyloid fibril surface.

The top scoring inhibitor candidate has two mutations, G2V and F4W. W4 formed hydrophobic interactions with hydrophobic residues L, M and V at the glycine groove. The other hydrophobic residue of the peptide, F8, is located between the N-terminus and C-terminus (see Fig. 4 (b) side view) of the beta sheets where it forms hydrophobic interactions with valines V40 of Aβs4 and Aβs5 and the phenylalanines F3 of Aβs2-4. The carbonyl of W4 and the amine of G6 form hydrogen bonds with the V39 backbone of Aβs5, which is located next to the Glycine groove. Hence, the inhibitor candidate grabs the fibril in a pincer-like form via W4 and F8, and further attaches via hydrogen bonds in the middle.

The second highest scoring inhibitor candidate, RGTFEGRF, is the K7R mutant. Here, F4 of the peptide is positioned in the glycine groove of the protein. The backbone carbonyl of F4 forms hydrogen bonds with the amines of two consequitive glycines residues G37 and G38 of Aβs5 on the fibril surface, one glycine residue at a time. Moreover, a hydrogen bond between R7 and the backbone of a phenylalanine F20 of Aβs5 exist.

The third best scoring inhibitor candidate, RITFEIKF, contains two mutations, G2I and G6I. I6 and F8 form hydrophobic interactions with the methionines in the GxMxG motif of Aβs3 and Aβs4 and the closely located isoleucines I31 of Aβs3–5. R1 was observed to form direct and water mediated hydrogen bonds with D23 of Aβs5 located at the Aβs sheet uniaxial interface and the carboxyl of M35 of Aβ5. However, these hydrogen bonds were observed to be rather ephemeral, breaking and forming in our CMD simulation. The backbone nitrogens of F4 and E5 formed hydrogen bonds with carboxyl group of G33 of Aβs5.

To sum up, the reference peptide and the second and third top scoring inhibitor candidates anchored to the amyloid fibril with a single hydrophobic residue and a single charged residue. The top scoring derivative, on the other hand, anchored with two hydrophobic residues. Their binding characteristics revealed a few key points. It can be concluded that hydrophobic interactions are the primary factor that facilitates peptide binding to the amyloid fibrils. The secondary factor appears to be the hydrogen bonds. However, in many cases the formation of hydrogen bonds was a direct result of the close spatial arrangement of charged atoms due to hydrophobic effects. In line with our findings, previous studies have demonstrated that hydrophobic/aromatic and hydrogen bonding interactions play critical roles in binding to the fibril and inhibiting its growth.[29], [30], [31]

Interestingly, in our simulations each of the four peptides (reference and its top three scoring derivatives) bound to the edge of the amyloid fibril, where they simultaneously interacted with the surfaces both parallel and perpendicular to the fibril axis direction (see Fig.4). Experimental[32], [33], [34] and computational[35] studies suggested that amyloid fibril extension takes place by monomer additions to the fibril edges. Moreover, it has been shown that the edge of amyloid fibril is a binding region for some chemicals including ibuprofen [36], [37] and morin[29], which share similar aromatic moieties with our peptide derivatives. Similar results have also been observed for congo red binding to Sup-35[30]. We speculate that the binding pattern observed here (to both amyloid surfaces) may be an effective mechanism for inhibiting both the interaction between interior faces of protofilaments and the addition of monomers along the fibril direction.

The replacement of residues G2 and F4 in the reference peptide with larger hydrophobic residues V and W resulted in a less compact and more stretched conformation of the peptide. This in turn allowed residues in range 2–7 to interact with the fibril surface and resulted in stronger interactions. Similarly, binding strength increased by mutating G2 and G6 into Isoleucines. Hence, to obtain optimum binding properties, the interacting surface area has to be maximized while keeping residues which interact well with the fibril surface in the peptide sequence.

### Potential of Mean Force for Unbinding of the Peptides

**Figure 5:**
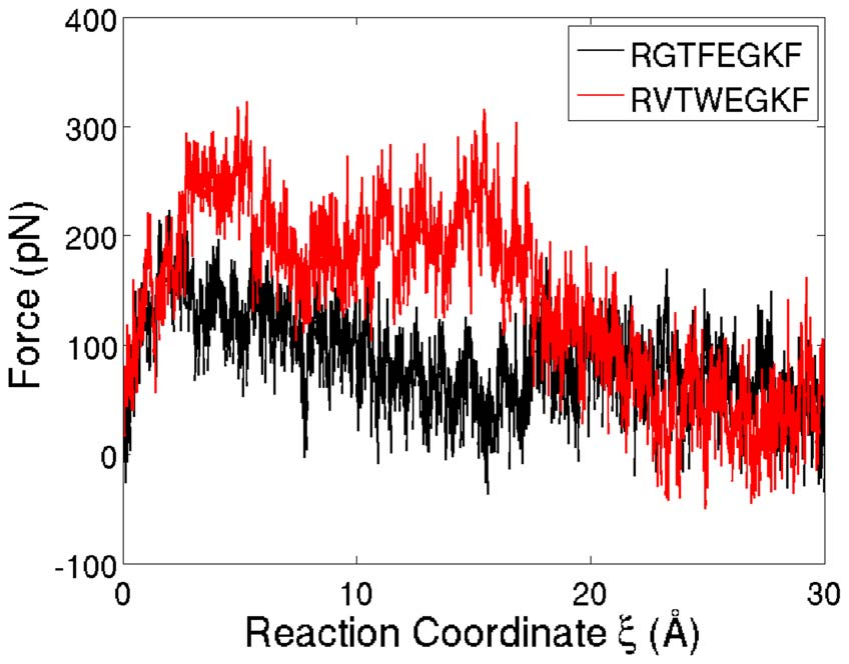
Forces Applied to Unbind the Peptides. The red curve shows the average force applied along the reaction coordinate 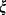 to unbind the top scoring inhibitor candidate whereas the black line shows the forces applied to unbind the reference peptide. Averages were taken over all SMD trajectories.

**Figure 6:**
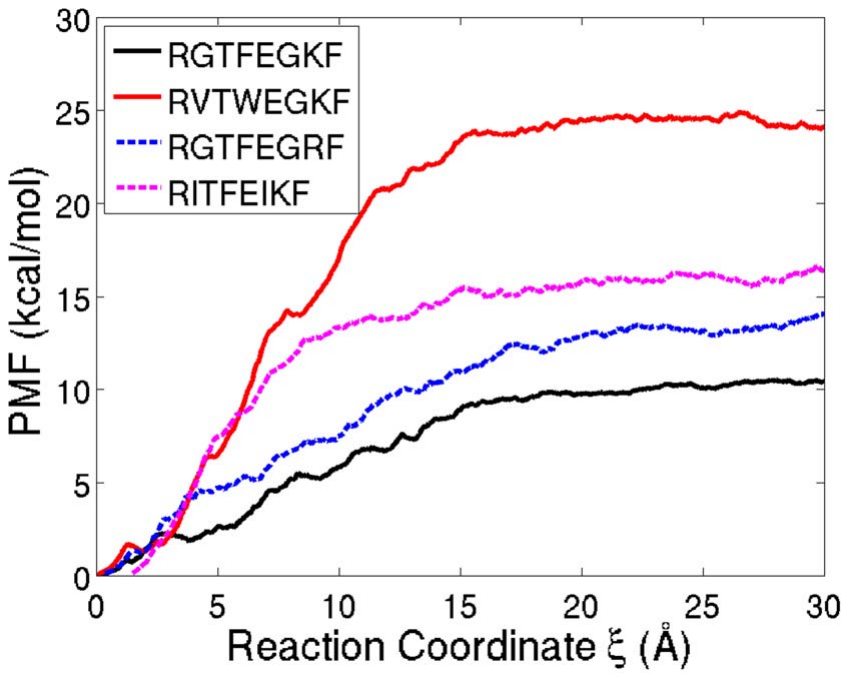
Potential of Mean Force for Unbinding. PMFs of the top scoring mutated peptides in Table 2 and the reference peptide with respect to the RC 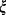.

The reference peptide and the 3 top scoring inhibitor candidates were selected for further investigation. For each of these peptides, 14 independent SMD simulations were performed. The ensemble averages of the external forces along the RCs of the reference peptide and top scoring inhibitor candidate (RVTWEGKF) are shown in Fig.5. The difference between the curves indicates that the unbinding of the reference peptide is easier than the top scoring peptide. In Fig.6, the PMFs for the unbinding of the three top scoring inhibitor candidates and the reference peptide are shown along their RCs. The PMFs predict a higher binding energy for all of the three derivative peptides in agreement with the Gold- and Chemscores. However, it has to be noted that the top scoring second and third peptides switched their rankings in the PMF.

## Conclusion

In this study a systematic and easy-to-use methodology to generate new peptide inhibitor candidates is introduced. The steps of the recursive cycles are well defined and straightforward to perform. Our methodology relies on widely used and proven techniques found in the literature. A final assesment of the candidate inhibitors using PMF strengthen the predictions made by the methodology. The methodology provides computationally promising inhibitor candidates, gives insight to the inhibitor binding problem, and delivers direction and a basis for further drug design analysis.

In order to analyze the unbinding process and investigate the quality of potential inhibitors under all atom MD simulations, the unbinding process of inhibitor candidates and the reference peptide were investigated using SMD. Due to computational load only the top ranking 3 inhibitor candidates of the ICL were chosen for further analysis, although other derivatives (Table 2) also gave better docking scores than the reference peptide. The SMD simulations showed that the designed peptides bind to the amyloid protofilament subunit with a higher affinity than the reference peptide and therefore affirmed the docking results for the selected three peptides. In the light of the docking and the SMD results, we strongly suggest that the designed inhibitor candidates are worth for further investigations.
